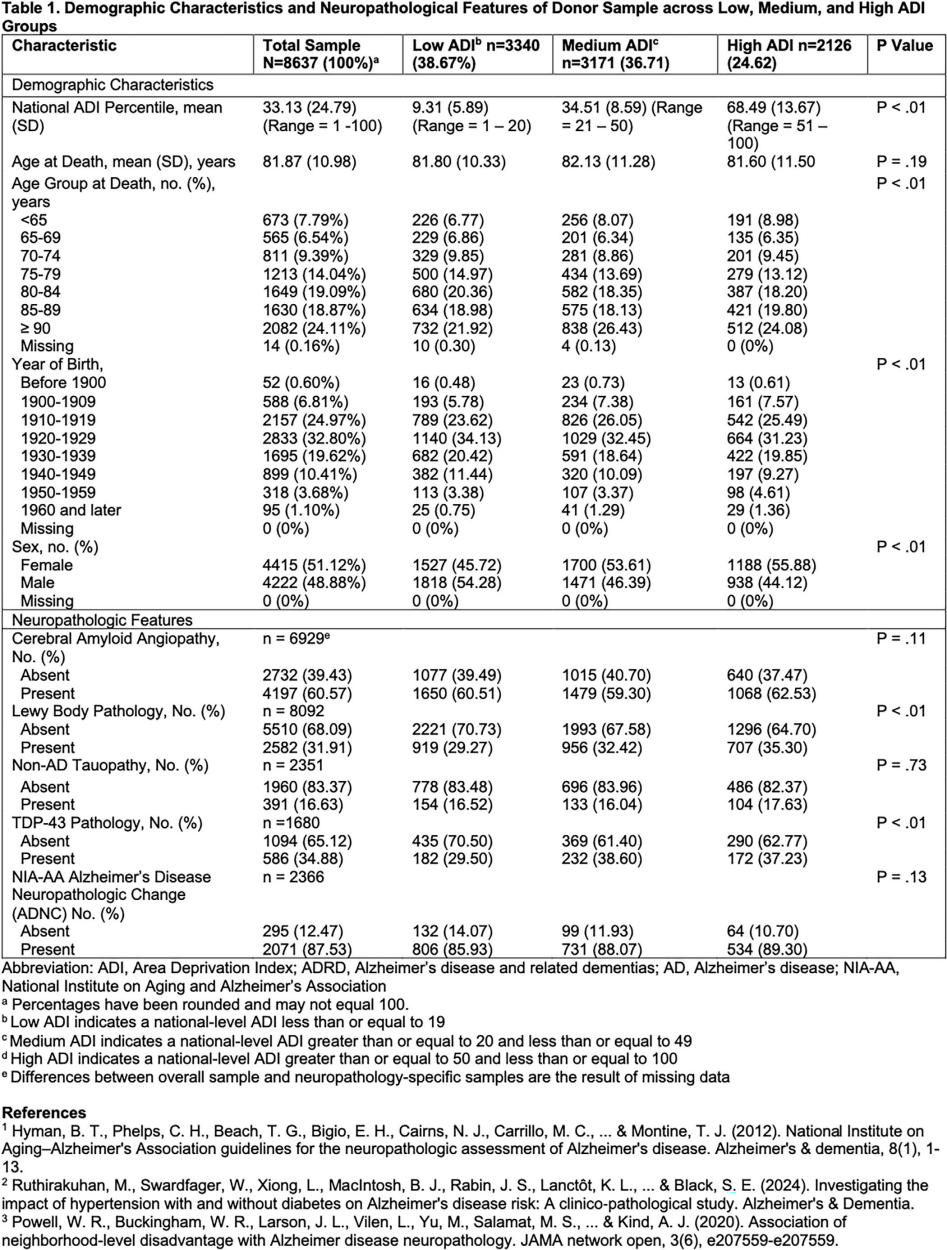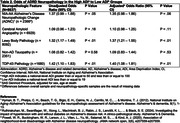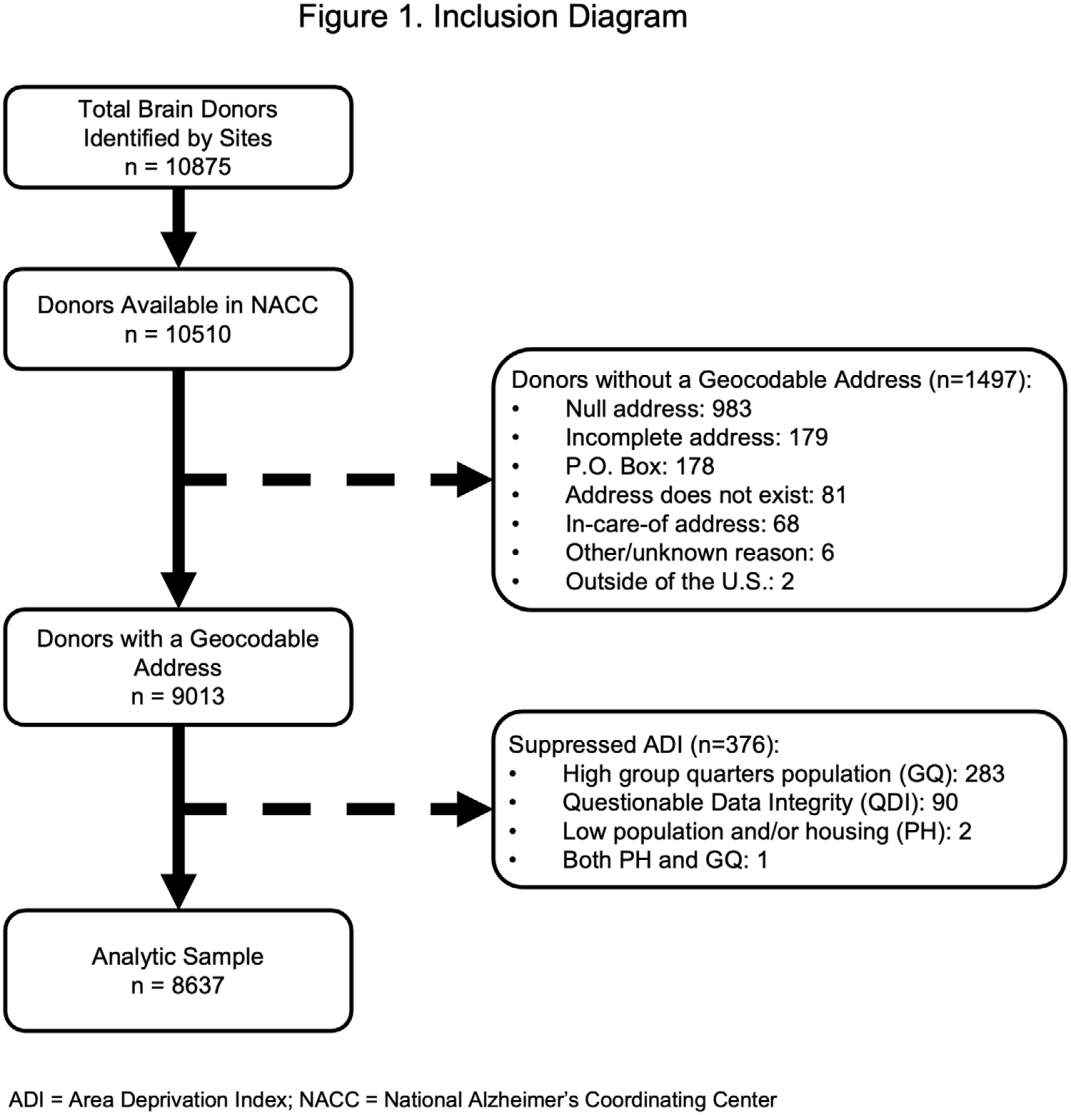# Adverse Social Exposome by Area Deprivation Index (ADI) and Alzheimer’s Disease and Related Dementias (ADRD) Neuropathology for a National Cohort of Brain Donors within the Neighborhoods Study

**DOI:** 10.1002/alz.095121

**Published:** 2025-01-09

**Authors:** Amy J.H. Kind, Barbara B. Bendlin, Sarah A. Keller, W. Ryan Powell, Amanda DeWitt, Yixuan Cheng, Luke Chamberlain, Brittney Lyons Boone, Megan J. Miller, Stacie M. Vik, Erin L. Abner, Michael L. Alosco, Liana G. Apostolova, Kelly M. Bakulski, Lisa L. Barnes, James R. Bateman, Thomas G. Beach, David A. Bennett, James B. Brewer, Carmen Carrion, Joshua Chodosh, Suzanne Craft, Raina Croff, Anthony Fabio, Sarah Tomaszewski Farias, Felicia Goldstein, Victor W Henderson, Thomas Karikari, Julia Kofler, Anna M. Kucharska‐Newton, Melissa Lamar, Serggio Lanata, Rebecca J Lepping, Jennifer H Lingler, Samuel N. Lockhart, Jonathan D Mahnken, Karyn Marsh, Oanh L. Meyer, Bruce L. Miller, Jill K Morris, Judith A. Neugroschl, Maureen K. O'Connor, Henry L Paulson, Richard J. Perrin, Aimee Pierce, Cyrus A. Raji, Eric M. Reiman, Shannon L. Risacher, Robert A. Rissman, Patricia Rodriguez Espinoza, Mary Sano, Andrew J. Saykin, Geidy E Serrano, David L Sultzer, Rachel A. Whitmer, Thomas Wisniewski, Randall Woltjer, Carolyn W. Zhu

**Affiliations:** ^1^ Center for Health Disparities Research, University of Wisconsin School of Medicine and Public Health, Madison, WI USA; ^2^ University of Kentucky, Lexington, KY USA; ^3^ Boston University School of Medicine, Boston, MA USA; ^4^ Indiana University School of Medicine, Indianapolis, IN USA; ^5^ University of Michigan School of Public Health, Ann Arbor, MI USA; ^6^ Rush University Medical Center, Chicago, IL USA; ^7^ Wake Forest University School of Medicine, Winston‐Salem, NC USA; ^8^ Banner Sun Health Research Institute, Sun City, AZ USA; ^9^ University of California, San Diego, La Jolla, CA USA; ^10^ Yale University, New Haven, CT USA; ^11^ NYU Alzheimer’s Disease Research Center, New York, NY USA; ^12^ Oregon Health & Science University, Portland, OR USA; ^13^ University of Pittsburgh, Pittsburgh, PA USA; ^14^ University of California, Davis, Sacramento, CA USA; ^15^ Emory University School of Medicine, Atlanta, GA USA; ^16^ Stanford University, Palo Alto, CA USA; ^17^ University of North Carolina Gillings School of Global Public Health, Chapel Hill, NC USA; ^18^ Memory and Aging Center, UCSF Weill Institute for Neurosciences, University of California, San Francisco, San Francisco, CA USA; ^19^ University of Kansas Medical Center, Kansas City, KS USA; ^20^ Wake Forest School of Medicine, Winston‐Salem, NC USA; ^21^ NYU Grossman School of Medicine, New York, NY USA; ^22^ University of California, Davis School of Medicine, Sacramento, CA USA; ^23^ University of California San Francisco, San Francisco, CA USA; ^24^ Icahn School of Medicine at Mount Sinai, New York, NY USA; ^25^ University of Michigan, Ann Arbor, MI USA; ^26^ Washington University in St. Louis, St. Louis, MO USA; ^27^ Washington University in St. Louis School of Medicine, St. Louis, MO USA; ^28^ Mount Sinai School of Medicine, New York, NY USA; ^29^ Indiana University, Indianapolis, IN USA; ^30^ University of California, Irvine, Irvine, CA USA; ^31^ University of California, Davis, Davis, CA USA; ^32^ New York University Grossman School of Medicine, New York, NY USA; ^33^ NIA‐Layton Oregon Alzheimer’s Disease Research Center, Oregon Health & Science University, Portland, OR USA; ^34^ Icahn School of Medicine, Mount Sinai Hospital, New York, NY USA

## Abstract

**Background:**

Adverse social exposome (indexed by high national Area Deprivation Index [ADI]) is linked to structural inequities and increased risk of clinical dementia diagnosis, yet linkage to ADRD neuropathology remains largely unknown. Early work from single site brain banks suggests a relationship, but assessment in large national cohorts is needed to increase generalizability and depth, particularly for rarer neuropathology findings.

**Objective:**

Determine the association between adverse social exposome by ADI and ADRD neuropathology for brain donors from 21 Alzheimer’s Disease Research Center (ADRC) brain banks as part of the on‐going Neighborhoods Study.

**Methods:**

All brain donors in participating sites with neuropathology data deposited at the National Alzheimer’s Coordinating Center (NACC) and identifiers for ADI linkage (N = 8,637; Figure 1) were included. Geocoded donor addresses were linked to time‐concordant national ADI percentiles for year of death, categorized into standard groupings of low (ADI 1‐19), medium (20‐49) and high (50‐100) ADI. Neuropathological findings were drawn from NACC and reflected standard assessment practices at time of donation. Logistic regression models, adjusted for sex and age at death, assessed relationships between high ADI and neuropathology findings.

**Results:**

Of the N = 8,637 brain donors (Table 1), 2,071 of 2,366 assessed (88%) had AD pathology by NIA‐AA criteria; 4,197 of 6,929 assessed (61%) had cerebral amyloid angiopathy; 2582 of 8092 assessed (32%) had Lewy body pathology; 391 of 2351 assessed (17%) had non‐AD tauopathy; and 586 of 1680 assessed (35%) had TDP‐43 pathology. 2,126(25%) were high ADI; 3,171(37%) medium ADI and 3,340(38%) low ADI with 51% female and average age at death of 81.9 years. As compared to low ADI donors, high ADI brain donors had adjusted odds = 1.35 (95% CI = 0.98‐1.86, p‐value = 0.06) for AD pathology; 1.10 (0.98–1.25, p = 0.11) for cerebral amyloid angiopathy; 1.37 (1.21–1.55, p<0.01) for Lewy body; 1.09 (0.83–1.44, p = 0.53) for non‐AD tauopathy; and 1.40 (1.08‐1.81, p = 0.01) for TDP‐43 pathology (Table 2).

**Conclusions:**

This first‐in‐field study provides evidence that the adverse social exposome (high ADI) is strongly associated with an increased risk of Lewy body, an increased risk of TDP‐43, and a trend towards increased AD pathology in a national cohort of brain donors.